# Methods for teaching postgraduate point-of-care ultrasonography: a systematic review

**DOI:** 10.1007/s11739-026-04535-3

**Published:** 2026-09-29

**Authors:** Marine Baumberger, Claudio Schneider, André Juillerat, Pierre Ritz, Radhika Sood, Stijn Bex, Christophe Marti, Jean Luc Reny, Olivier Grosgurin, Antonio Leidi

**Affiliations:** 1 Division of General Internal Medicine, Department of Medicine, Geneva University Hospitals, Geneva, Switzerland; 2 Division of Emergency Medicine, Department of Acute Care Medicine, Geneva University Hospitals, Geneva, Switzerland; 3 Department of General Internal Medicine, Inselspital, Bern University Hospital, University of Bern, Bern, Switzerland; 4 Division of Primary Care Medicine, Geneva University Hospitals, Geneva, Switzerland; 5 Division of Pneumology, Department of Medicine, Geneva University Hospitals, Geneva, Switzerland

**Keywords:** Point-of-care ultrasonography, Education, Teaching methods, Post-graduate, Randomized controlled trial

## Abstract

Point-of-care ultrasonography (POCUS) is increasingly used across multiple medical specialties, yet the impact of different teaching methods on learning outcomes remains unclear. Conduct a systematic review of randomized controlled trials (RCTs) comparing different methods of POCUS training and synthesize the available data. We searched MEDLINE, EMBASE, Cochrane, ERIC, and PsycINFO without language or date restrictions. Eligible studies were RCTs evaluating POCUS teaching methods for physicians. Studies focusing on pre-graduate learners, transesophageal or transvaginal ultrasound and procedural guidance were excluded. Records were screened and data were extracted by independent reviewers. Methodological quality assessment was performed using the Modified Medical Education Research Study Quality Instrument. The study protocol was prospectively registered in the international database of PROSPERO (CRD42024512650). Thirty-seven RCTs including 1,571 participants were identified. Interventions were grouped as simulation-based training (ten studies), web-based or video-assisted learning (ten studies), variations in training intensity or structure (eight studies), tele-mentoring (four studies), and other methods (five studies), mostly in comparison with traditional classroom teaching. Across most trials, interventions produced theoretical and practical skills without significant difference to traditional classroom models. Superiority was mainly observed when the intervention arm received greater training intensity or longitudinal supervision. Most studies used heterogeneous, non-standardizable assessments, precluding meta-analysis. For non-procedural postgraduate POCUS, several sustainable educational alternatives exist and may allow for similar competence when compared to traditional teaching. Training intensity, supervision, and longitudinal exposure may be more important determinants of learning outcomes than the teaching modality itself. Cognitively enriched formats may provide additional benefits. Further research is needed to define the optimal structure of POCUS curricula. Only one study assessed patient-centered outcomes, limiting conclusions regarding the clinical impact of educational interventions.

**Supplementary Information:**

The online version contains supplementary material available at https://doi.org/10.1007/s11739-026-04535-3.

## Introduction

Point-of-care ultrasonography (POCUS) has become a routine tool in hospital medicine, allowing clinicians to address focused clinical questions directly at the patient’s bedside [1]. Current evidence supporting its use is well established, with studies showing improvements in diagnostic accuracy, patient management, and cost-effectiveness [2, 3]. However, the value of POCUS depends heavily on the operator’s technical skills and interpretative expertise. Training physicians to achieve POCUS proficiency requires substantial time and resources. Although several professional societies have published recommendations for POCUS training, these guidelines are largely based on expert opinion rather than established evidence [4, 5]. As a result, educational approaches and curricula vary widely across institutions, reflecting the absence of standardized and validated teaching frameworks.

No review has yet comprehensively synthesized randomized controlled trials (RCTs) evaluating POCUS training methods for postgraduate physicians across different organ assessments. As POCUS continues to expand across medical specialties, a rigorous, evidence-based summary is warranted to guide further development of effective and clinically meaningful training programs.

The objective of this systematic review is therefore to identify RCTs comparing different methods of POCUS training and synthesize the available data on the effectiveness of different educational strategies used to teach POCUS during postgraduate training.

## Methods

This systematic review was prepared in accordance with the Preferred Reporting Items for Systematic Review and Meta-Analysis (PRISMA) guidelines [6] and was registered in the International Prospective Register of Systematic Reviews (PROSPERO) on 13.02.2024 before conducting the search and analyzing data (PROSPERO registration number: CRD42024512650). Five databases were used: MEDLINE via PubMed, EMBASE via OVID, Cochrane, ERIC, and PsycINFO. The PRISMA checklist is provided in Online Resource 1.

Each of the two concepts, “ultrasonography” and “education”, was searched using free-text and thesaurus terms (e.g., MeSH in MEDLINE), and the two were combined using AND. The search queries (available in Online Resource 2) were constructed in collaboration with a librarian of the University of Geneva. No language or date restrictions were applied. Records were managed in the reference tool EndNote X9 (Clarivate Analytics, Philadelphia, United States), and the records were uploaded to the Internet-based software program Covidence (Veritas Health Innovation Ltd., Melbourne, Australia, 2024) was used, where duplicates were removed and articles screened. The search was completed on February 16, 2024, and updated prior to the final analysis on May 22, 2025. The reference lists of eligible articles were screened to identify relevant studies not retrieved by the primary search.

### Eligibility criteria

All randomized controlled trials focusing on methods for teaching POCUS to physicians in postgraduate training were included. The following were excluded: non-randomized studies, studies involving ultrasonography training for specialists who routinely use it in clinical practice (e.g., radiologists, cardiologists, obstetricians), studies focused on pre-graduate teaching, studies focused only on procedural guidance, and studies focused on transesophageal echocardiography or transvaginal ultrasonography. Moreover, abstracts without available full text were excluded due to insufficient methodological details. No language or date restrictions were applied. More details are available in Table S1 from Online Resource 3.

### Study selection, data extraction and synthesis

All records identified through searches were imported into the Covidence reference software, where duplicates were removed semi-automatically and manually by eight authors (MB, AL, CS, AJ, PR, RS, CM, SB).

Titles and abstracts were screened independently by two reviewers. Full-text studies were retrieved and independently assessed for inclusion by two authors. In cases of disagreement, disagreements were resolved by a third reviewer (AL or OG). Although no formal calibration exercise was performed, all reviewers were trained on the study protocol prior to initiating the review process.

A standardized data extraction form adapted from the Cochrane form was developed by three reviewers (AL, MB, CS) prior to data extraction. Data was extracted independently by two authors (MB, CS). Meta-analysis was not possible due to the heterogeneous teaching and assessment methodologies, thus an integrative approach to data synthesis was employed.

Risk of bias was assessed using the Modified Medical E6ducation Research Study Quality Instrument (MMERSQI), a tool adapted in 2023 [7] through a Delphi consensus to address several limitations of the original validated tool (MERSQI) for evaluating the quality of experimental and observational studies in medical education. The modified instrument was applied independently by two authors (MB, CS). Disagreements were resolved by consensus or by a third author when needed. The MMERSQI score can be found in Online Resource 4.

## Results

**Fig. 1:**
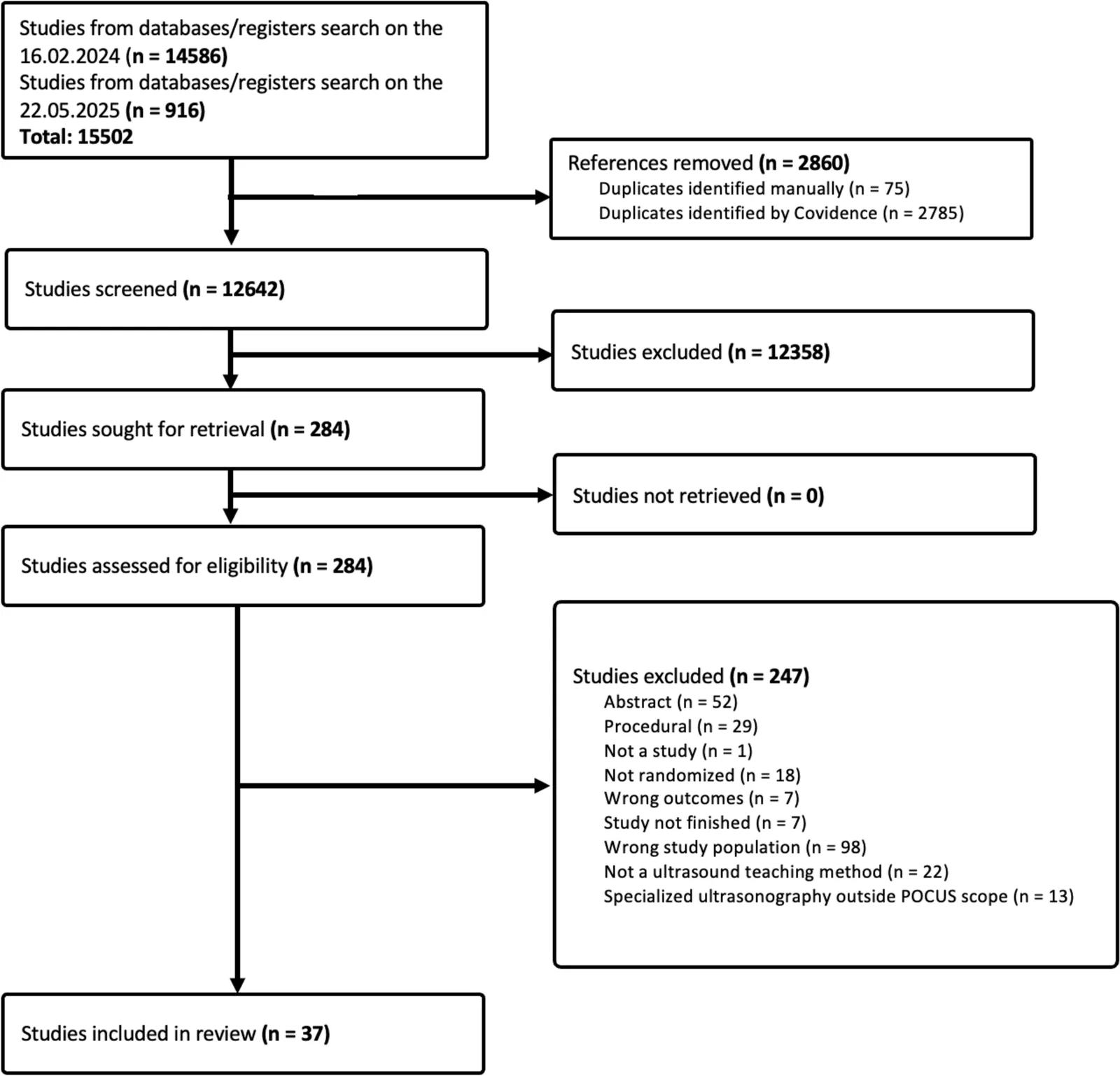
PRISMA flow diagram

The initial systematic search was conducted on February 16, 2024, identifying 14,586 records across the 5 databases. An updated search performed on 22 May 2025, yielded an additional 916 records. After removing duplicates, 12,642 titles and abstracts were screened, of which 12,358 were excluded. A total of 284 full-text articles were then assessed for eligibility, and 37 studies were retained for the final synthesis. The complete selection process is illustrated in the PRISMA flowchart in Fig. 1.

### Study characteristics

In total, 1,571 participants were included across 37 RCTs, which were categorized into 5 groups based on their educational characteristics. The most frequent teaching methods, summarized in Table 1, were simulation-based training (ten studies) and web-based learning (ten studies), followed by variations in training intensity (eight studies), other methods (five studies), and tele-mentoring (four studies). The comparator in most studies was traditional teaching (i.e., mainly classroom with theoretical and practical components).

**Table 1:** Summary of teaching methods

Teaching method	Description	Studies
Simulation-based	Using a simulator	[8–17]
Web-based	Online modules, videos, apps, or e-learning platforms	[18–27]
Training intensity	Variations in duration, supervision, or training volume	[28–35]
Other methods	Alternative educational approaches	[36–40]
Tele-mentoring	Remote supervision or real-time guidance during training	[41–44]

### Type of POCUS

Across the 37 studies, Focused Assessment with Sonography for Trauma (FAST), including its extended variant (eFAST), was the most frequently investigated application (ten studies), followed by cardiac ultrasound (seven studies) and lung ultrasound (five studies). Abdominal ultrasound and shock-assessment protocols (Rapid Ultrasound for Shock and Hypotension, RUSH) were each examined in two studies, while ocular ultrasound appeared in one (see Table S2 in Online Resources 3). Ten studies focused on multiple POCUS applications.

### Training duration

**Fig. 2:**
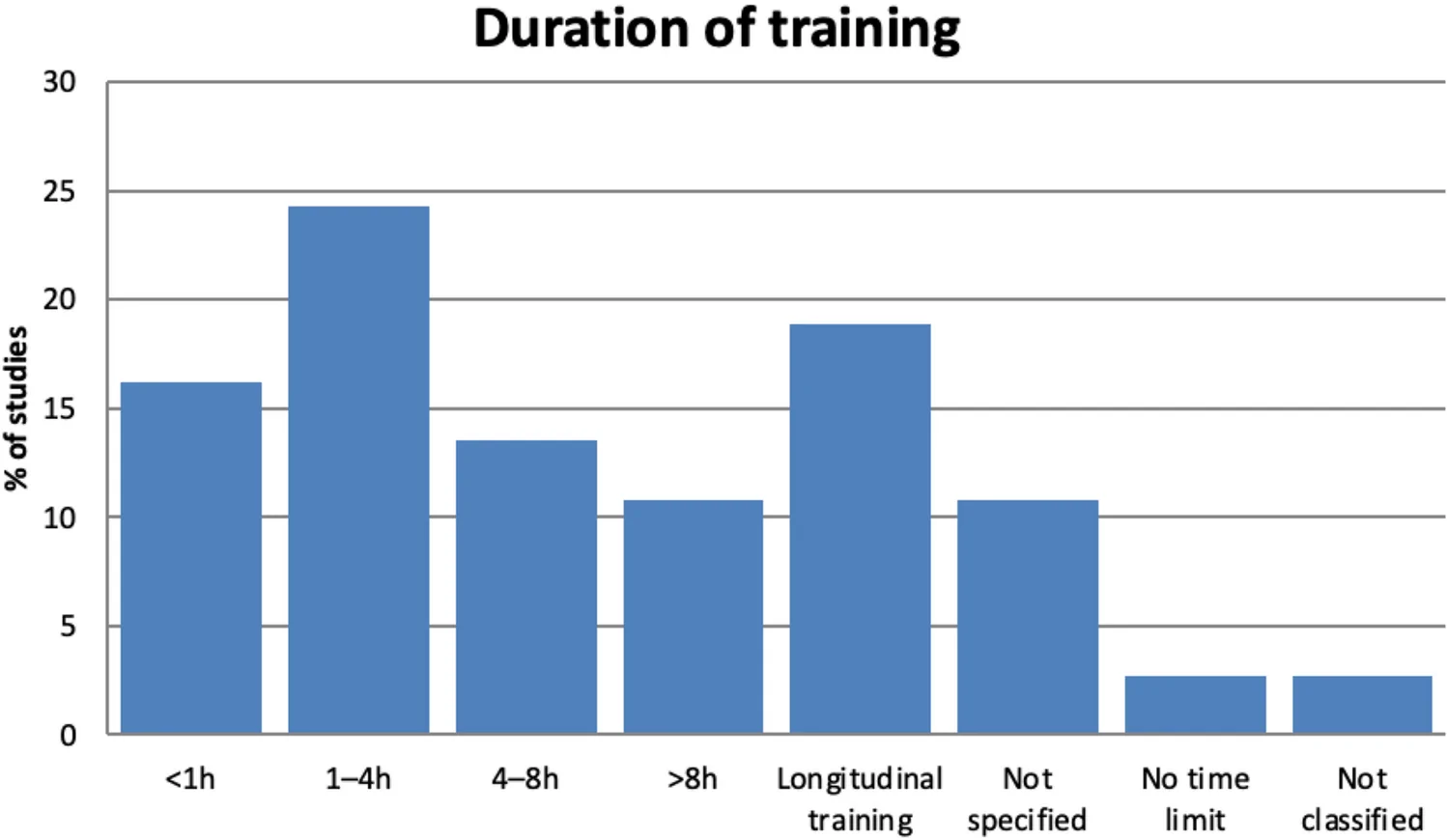
Training duration. Not specified: not specified in the study. Not classified: could not be classified in one of the specified categories [40]

Total training duration and format varied across the 37 studies (Fig. 2). Most studies (20/37, 54.1%) reported a total training duration of less than 8 h [8–11, 13, 15, 16, 19–27, 30, 31, 33, 44]. Four studies implemented intensive single-day formats lasting ≥ 8 h (4/37, 10.8%) [18, 29, 37, 39] and seven studies adopted a longitudinal format with repeated training sessions or follow-up over several weeks to months (7/37, 18.9%) [12, 14, 17, 28, 32, 34, 38].

### Previous experience

Previous ultrasound (US) experience was reported by participants in 43.2% (16/37) of the included studies [8, 10, 13, 15, 18, 22, 24, 25, 28, 31, 34, 39–43], whereas in 32.4% (12/37) of the studies [9, 11, 12, 16, 19, 20, 23, 26, 27, 29, 30, 36], the participants were novices, and in 24.3% (9/37) of the studies [14, 17, 21, 32, 33, 35, 37, 38, 44], this information was not specified. More details on participants’ characteristics and assessment tools are available in Online Resource 3 (section “Assessment”) and Table S7.

### Risk of bias in the studies

All 37 randomized controlled trials were assessed using the Modified Medical Education Research Study Quality Instrument (MMERSQI). MMERSQI scores ranged from 48 to 89 points out of 100, with a mean score of 69.2 (SD = 10.7) and a median of 71.0, with a higher score indicating a lower risk of bias. Most studies (*n* = 31, 83.8%) achieved scores ≥60, and seven studies (18.9%) obtained scores ≥80. Interrater agreement between the two independent reviewers for the MMERSQI score was substantial (weighted Cohen’s kappa coefficient of 0.74) (Table 2).

**Table 2:** Modified MERSQI score

Author’s name	Modified MERSQI points (nb)
Simulation-based	
Salen et al. 2001 [8]	53
Edrich et al. 2014 (heart) [9]	79
Paddock et al. 2015 [10]	64
Wilkinson et al. 2016 [11]	74
Silva et al. 2016 [12]	64
Juo et al. 2017 [13]	61
Frère et al. 2021 [14]	64
Neelankavil et al. 2021 [15]	76
Pietersen et al. 2021 [16]	87
Blanchetière et al. 2025 [17]	84
Web-based	
Platz et al. 2010 [18]	64
Edrich et al.2016 [19]	72
Nilsson et al. 2017 [20]	89
Haskins et al. 2018 [21]	73
Tomasi et al. 2018 [22]	49
Monti et al. 2020 [23]	66
Soon et al. 2020 [24]	72
Isa et al.2021 [25]	89
Kang et al. 2022 [26]	84
Morales-Castro et al. 2025 [27]	63
Training intensity	
Leidi et al. 2025 [28]	89
Noble et al. 2007 [29]	58
Ramsingh et al. 2014 [30]	61
Todsen et al. 2016 [31]	81
Matthews et al. 2020 [32]	71
Smith et al. 2021 [33]	69
Suzuki et al. 2022 [34]	67
Liu et al. 2025 [35]	59
Other methods	
Campanella et al. 2004 [36]	48
Lai et al. 2020 [37]	76
Buesing et al. 2021 [38]	77
Hofer et al. 2022 [39]	74
Baum et al. 2023 [40]	63
Tele-mentoring	
Mao et al. 2023 [41]	59
Gillon et al. 2024 [42]	61
Shen et al. 2024 [43]	60
Kim et al. 2025 [44]	76

### Data synthesis

The studies included in this review are described below, grouped by the principal training method being assessed.

### Simulation-based training

Nine randomized controlled studies [8–17] compared simulation-based POCUS training with traditional teaching methods and one [15] compared simulation-based POCUS versus video-only teaching. Further details regarding individual study characteristics and outcomes can be found in Table S3 of Online Resource 3.

Among them, five studies reported no significant difference between simulation-based and traditional teaching methods for either theoretical or practical performance [8–12], while one study showed that both methods outperformed a no-training control group [16]. Neelankavil et al. [15] reported a higher written and practical performance in the simulation-based group; however, this group performed hands-on scanning, whereas the comparison group did not. Juo et al. [13] found that written test improvements were similar in both groups, while the live-patient group achieved a higher practical performance. It is noteworthy that in this trial, a low-fidelity simulator was used, a limitation that may explain the inferior acquisition skills observed in the simulator group. Two studies reported higher practical performances among learners who received simulation-based training [14, 17]. However, in both cases, the simulation groups also received substantially more total practice through repeated sessions [14] or structured clinical exposure combined with simulation [17], which may have influenced the results.

### Web-based training

Nine randomized studies [18–26] compared web-based or video-assisted POCUS instruction with traditional teaching formats and one [27] compared video-assisted education with simulation-based teaching. Further details regarding individual study characteristics and outcomes are reported in Table S4 of Online Resource 3.

Across these studies, no significant difference in theoretical or practical performance was reported between online or video-based learning and traditional teaching methods [18–27]. Most studies delivered a structured web-based or e-learning curriculum [18, 19, 21–26], while Morales-Castro et al. [27] provided the same theoretical content to both groups but offered additional instructional videos exclusively to the intervention group. Mobile app-based self-directed training [20] was compared with textbook-guided learning for eFAST acquisition. Both groups trained independently for 2 h, with no significant differences observed in theoretical scores or in practical performance as assessed by the Objective Structured Assessment of Ultrasound Skills (OSAUS) scale. Soo-Yeon Kang et al. [26] evaluated an online ocular POCUS program delivered without hands-on training and reported non-inferior practical and theoretical performance compared with traditional teaching. Edrich et al. [19] and Tomasi et al. [22] both investigated lung POCUS, with intervention groups receiving video-based instruction combined with self-scanning components, resulting in similar practical and theoretical performance [19] and satisfaction [22] to conventionally trained groups.

### Tele-mentoring

Three randomized studies [41, 42, 44] compared remote POCUS instruction delivered via tele-mentoring technologies with traditional in-person training, and none reported significant differences between the two methods. One study [43] compared remote POCUS instruction with web-based training, and reported higher theoretical and practical scores in the tele-mentoring group. Notably, the web-based training in this study did not include any hands-on component, which may explain the superiority in the tele-mentoring arm that included hands-on practice by design. Further details regarding individual study characteristics and outcomes can be found in Table S5 of Online Resource 3.

Beyond performance outcomes, tele-mentoring was also associated with a more favorable learner experience in some trials: one study reported higher participant satisfaction in the tele-mentoring group [43], while another showed that learners engaged in more educational interactions and reported lower mental demand, effort, and frustration, as measured by workload scales [44].

### Training intensity

Eight randomized studies [28–35] compared POCUS training interventions differing in duration, structure, or intensity of supervised practice. Further details regarding individual study characteristics and outcomes can be found in Table S6 of Online Resource 3.

One study, comparing no training at all to structured education, showed a clear advantage for the trained group [31]. This benefit was visible on a practical test, where trained participants demonstrated markedly higher diagnostic accuracy than those with no education. Among studies comparing theoretical-only instruction to training that included hands-on components, two trials demonstrated that adding practical exposure resulted in better performance than theory alone [29, 30]. Smith et al. [33] demonstrated that mastery-based instruction, incorporating iterative feedback and repeated testing until proficiency, was superior to a traditional lecture-based approach. The mastery-learning group achieved significantly higher scores during the early training phases, and both groups reached proficiency after the cross-over. The study by Liu Q et al. [35] showed that a multimodal, problem-oriented teaching strategy combining visualization, mind mapping, and hands-on practice significantly outperformed standard classroom teaching in both theoretical knowledge and practical skills, while also improving learner engagement, satisfaction, and self-directed learning.

Two other studies evaluating enhanced or longitudinal supervision found that structured follow-up practice and repeated supervised scanning sessions led to further improvements in technical skills, autonomy, and long-term retention [28, 32]. In contrast, another study found no additional benefit of extra training [34]. However, in this study, the intervention group benefited from 8 h of additional training on top of an already extensive baseline curriculum, which lasted 6 months and included self-paced online learning modules, in-class lectures, and hands-on training at the bedside.

### Other methods

Five randomized studies [36–40] compared alternative POCUS teaching methods. Further details regarding individual study characteristics and outcomes can be found in Table S6 of Online Resource 3.

In one study, participants using an artificial intelligence (AI)-assisted handheld ultrasound were compared to those using an unguided handheld probe over a 2-week practice period [40]. Participants in the AI group achieved faster acquisition of cardiac views and produced higher-quality images (no AI support was available during evaluation), and the confidence levels of participants were similar between groups. In line with these results, another study found that providing a handheld ultrasound device for practice without supervision did not increase scan frequency or improve proficiency [38].

One study [39] compared peer-assisted learning by trained student instructors to physician-led instruction. Both groups completed more than 17 h of theoretical and hands-on training, with comparable performance on the Objective Structured Clinical Examination** (**OSCE) and didactic competence.

Gamified teaching using team-based quizzes and points was evaluated to supplement RUSH protocol instruction [37]. Theoretical, practical, and retention test outcomes were comparable between the gamified and traditional groups, with higher reported motivation in the gamified arm.

Campanella et al. [36] assessed the addition of dissected bovine hearts to standard cardiac POCUS teaching for anatomical visualization. The intervention was limited to image recognition without any practical scanning component. Adjusted post-test theoretical scores showed no significant difference with the control group.

## Discussion

Most included studies evaluating simulation-based education reported no significant differences in learning outcomes compared with live-patient teaching. Differences were observed only when a low-fidelity simulator was used [13] or when the training load differed substantially [14, 15, 17]. Consistently, a scoping review by Moses et al. [45] reported that simulators and human models appear to be equivalent for teaching knowledge and image interpretation, whereas human models may be superior for teaching image acquisition. Simulators often present idealized images lacking the anatomical and physiological variability seen in real patients, which may explain the observed difference. Simulators nonetheless offer important advantages: they allow repeated practice, exposure to pathological findings absent in healthy volunteers, and training without involving or potentially resulting in discomfort for real patients [46, 47]. In summary, simulation appears to be a safe and reliable substitute for live-patient teaching in many contexts but cannot fully replace real clinical scanning.

Most studies comparing web-based or video-assisted teaching to traditional instruction found no significant differences in knowledge acquisition or practical performance [18–27]. This is consistent with a systematic review of e-learning in POCUS among students [48], which found that web-based POCUS teaching is overall comparable to traditional methods for thoracic, FAST, and musculoskeletal POCUS, while also demonstrating similar levels of learner satisfaction.

Even when online groups received no supervised hands-on practice, such as in ocular [26] or lung POCUS [19, 22], knowledge outcomes showed no significant difference to those of in-person groups, although learners often reported lower confidence or satisfaction. These modalities (ocular and lung POCUS) are known to have shorter learning curves, which may partly explain these findings. However, a recent scoping review [49] showed that online teaching-only is effective for image interpretation but has mixed results for image acquisition skills.

In our systematic review, most studies evaluating tele-mentoring reported no significant differences in performance outcomes compared with traditional in-person teaching, while tele-mentoring appeared to outperform theoretical-only learning without practical training. These findings align with a scoping review on point-of-care tele-ultrasound [50], which concluded that real-time tele-mentoring is feasible, acceptable, and supports safe image acquisition in settings lacking on-site experts. Tele-mentoring has also been highlighted as a recommended best practice, particularly for low-resource settings, by a scoping review on POCUS training in underserved contexts [51]. This reinforces the idea that tele-supervision may represent a scalable and sustainable solution to expand access to high-quality ultrasound education, especially when local expertise is limited.

Several studies addressed the influence of training duration and structure on POCUS skill acquisition. Longitudinal designs involving repeated scanning and ongoing supervision [28, 32] suggested better competency compared with shorter formats. These findings suggest that fragmented practice and iterative feedback, rather than isolated training events, may play a role in consolidating both technical performance and confidence over time. This pattern aligns with broader evidence from ultrasound education research. A recent scoping review [45] concluded that longitudinal curricula yield stronger learning outcomes, particularly technical skills and diagnostic accuracy, although they require greater resource investment. Additional evidence from a systematic review on competency retention [52] demonstrated that longer courses resulted in substantially better long-term retention. Overall, extended and longitudinal training formats appear to be more favorable for durable POCUS skill acquisition than short, isolated interventions.

Several studies explored whether modifying the pedagogical approach itself could enhance POCUS learning [33, 35, 37]. Overall, cognitive enriched approaches [35] seemed to improve learning whereas motivational formats like gamification appeared to increase engagement without consistently improving skills [37]. Mastery learning [33] enabled learners to reach proficiency milestones earlier.

A previous systematic review [52] suggested that the most effective learning environments may be those that combine multiple complementary strategies. Together, these findings highlight that alternative pedagogical methods could influence POCUS learning, either by enhancing cognitive processing or boosting learners’ engagement. However, the existing evidence remains limited, and further research is needed to determine which educational strategies most effectively complement or improve standard instructional models.

This systematic review has some limitations. First, the findings apply exclusively to postgraduate physicians, and more specialized ultrasound applications, such as transvaginal or transesophageal ultrasonography, were excluded, limiting the generalizability to other POCUS contexts and applications. Second, all but one study did not assess patient-centered clinical outcomes. Consequently, it remains unclear whether the observed improvements in educational performance translate into meaningful clinical benefits or improved patient care.

Third, methodological appraisal was conducted using the Modified MERSQI score. This tool, while derived from the validated original MERSQI, needs additional validation in larger studies. The resulting scores should be interpreted cautiously and primarily as comparative indicators of methodological quality rather than definitive measures of risk of bias. Fourth, considerable heterogeneity was observed across trials regarding learner backgrounds, POCUS applications, training intensity, assessment methods, efficacy outcomes, and follow-up intervals. This variability limited direct comparability between studies and precluded quantitative meta-analysis. It also reflects the broader lack of standardized POCUS curricula and validated outcome measures in postgraduate education. Lastly, most studies assessed only short-term educational outcomes, with minimal evaluation of long-term skill retention, which is particularly relevant for psychomotor skills known to fade over time.

This systematic review has several strengths. First, the comprehensive screening of a substantial number of references, combined with the inclusion of multiple databases, underscores the methodological rigor and sensitivity of the search strategy, thereby enhancing the completeness and validity of the review. Second, to our knowledge, this is the first systematic review focusing on RCTs on postgraduate POCUS education across a broad range of clinical applications. Third, the comprehensive scope of POCUS applications covered allows for the exploration of training effectiveness across diverse clinical contexts rather than a single organ system or specialty.

In conclusion, for postgraduate non-procedural POCUS, several educational modalities reported no significant differences compared to traditional teaching. Differences in skill retention seemed to be mostly observed in studies where the training load differed between groups. Therefore, training intensity, supervision, and longitudinal exposure may be more important determinants of learning outcomes than the teaching modality itself. Importantly, some of these alternatives appear to be more time-efficient and less resource-intensive for instructors, which is a critical consideration in the context of the growing demand for POCUS training and the limited availability of qualified instructors [53]. Future work should explore longitudinal training models, as literature suggests that skill retention may require repeated practice rather than isolated short workshops. Emerging technologies, including AI-assisted ultrasound and smart handheld devices, also represent promising future directions to support skill acquisition. Research should further prioritize standardized assessments, long-term follow-up, and patient-centered outcomes to better understand how educational gains translate into clinical impact and therefore define optimal postgraduate POCUS curricula.

## Supplementary Information

Below is the link to the electronic supplementary material.

Supplementary file1 (PDF 148 KB)

Supplementary file2 (DOCX 16 KB)

Supplementary file3 (DOCX 171 KB)

Supplementary file4 (PDF 488 KB)

## Data Availability

The data supporting the findings of this systematic review are derived from the published studies included in the review. No new datasets were generated or analysed during the current study.
