# Supplementary material for: Methods for teaching postgraduate point-of-care ultrasonography: a systematic review
Source: Intern Emerg Med. 2026 Sep 29;21(8):3101–10. doi: 10.1007/s11739-026-04535-3 (PMC13751701; doi:10.1007/s11739-026-04535-3)
Supplement: Supplementary file 2 [file 11739_2026_4535_MOESM4_ESM.pdf]

Methods for teaching postgraduate point-of-care ultrasonography: a systematic review

Online resource 4

Dr Marine Baumberger[1,2], Dr Claudio Schneider[1], Dr André Juillerat[1], Dr Pierre Ritz[3], Dre Radhika Sood[4], Dr Stijn Bex[1,2], Dr Christophe Marti[1], Prof. Jean Luc Reny[1], Dr Olivier Grosgurin[1,2]*, Dr Antonio Leidi[1,2]*

[1]Division of General Internal Medicine, Department of Medicine, Geneva University Hospitals, Geneva, Switzerland

[2]Division of Emergency Medicine, Department of Acute Care Medicine, Geneva University Hospitals, Geneva, Switzerland

[3]Division of Primary Care Medicine, Geneva University Hospitals, Geneva, Switzerland.

[4]Division of Pneumology, Department of Medicine, Geneva University Hospitals, Geneva, Switzerland.

Corresponding author: Marine.baumberger@hug.ch

*These authors contributed equally to the manuscript

Table 2 The final Modified MERSQI; items after Deplhi-1, scores after Delphi-2

Domain	MMERSQI Item	Points	Score		
			Each	Total	Max
Study design	1. Study design: (Pls. select one)				
	a. Single group cross-sectional or single group post-test only	7			23
	b. Single group pre-test & post-test	9			
	c. Nonrandomised, 2 groups	10			
	d. Randomised controlled trial with high risk bias[a]	11			
	e. Randomised controlled trial with moderate risk bias[a]	16			
	f. Randomised controlled trial with low risk bias[a]	23			
Sampling	2. Is there a power calculation (***sufficient statistical power***) for sample size?				10
	a. No	0			
	b. Yes	3			
	3. Are detailed participant characteristics for each arm reported?				
	a. No	0			
	b. Yes	3			
	4. Response rate, %: (Pls. select one)				
	a. Not reported	0.5			
	b. < 50	1			
	c. 50-74	2			
	d. > 75	4			
Setting	5. Institutions studied: *(Pls. select one)*				8
	a. Single centre	5			
	b. Multi centre no further specification	5			
	c. Multi centre with specification but not appropriate / balanced / complementary	5			
	d. Multi centre with appropriate and balanced / complementary	8			
Type of data	6. Type of data				11
	Assessment by participants	4			
	Objective measurement (*Pls. select one)*				
	a. Knowledge test (e.g. recall type questions)	6			
	b. Applied knowledge test (e.g. analysis and problem-solving type questions)	8			
	c. Skills	11			
Validity of evaluation instrument	7. Internal structure:				15
	a. Not applicable				
	b. Not reported	0			
	c. Reported	5			
	8. Content:				
	a. Not applicable				
	b. Not reported	0			
	c. Reported	5			
	9. Relationships to other variables:				
	a. Not applicable				
	b. Not reported	0			
	a. Reported	5			

Table 2 (continued)

Domain	MMERSQI Item	Points	Score		
			Each	Total	Max
Data analysis	10. Appropriateness of analysis:				17
	a. Inappropriate for study design or type of data	0			
	b. Appropriate for study design, type of data	9			
	11. Complexity of analysis (***if appropriate for study design***): *(Pls. select one)*				
	a. Descriptive analysis only	4			
	b. Simple inferential statistics	4			
	c. Modelling and more complex analysis	8			
Outcomes	12. Outcomes				
	Satisfaction, attitudes, perceptions, opinions, general facts	7			16
	Knowledge, measured by: (Pls. select one)				
	**a. *Low fidelity simulation or paper-based assessments***	9			
	**b. *High fidelity simulation***	12			
	Skills measured by:				
	**a. *Low fidelity simulation or paper-based assessments***	8			
	**b. *High fidelity simulation***	12			
	Behaviours in clinical environment	13			
	Patient/health care outcome	16			
Total Possible score				Min 23.5	Max 100

a Risk of bias judgment based on: sequence generation, blinding & allocation concealment. For more details, please see Additional file 2 Cochrane Risk of Bias Tool for Randomized Controlled Trials
