# Supplementary material for: Methods for teaching postgraduate point-of-care ultrasonography: a systematic review
Source: Intern Emerg Med. 2026 Sep 29;21(8):3101–10. doi: 10.1007/s11739-026-04535-3 (PMC13751701; doi:10.1007/s11739-026-04535-3)
Supplement: Supplementary file 3 [file 11739_2026_4535_MOESM2_ESM.docx]

**eMethods for teaching postgraduate point-of-care ultrasonography: a systematic review**

**Online resource 2**

Dr Marine Baumberger^1,2^, Dr Claudio Schneider^1^, Dr André Juillerat^1^, Dr Pierre Ritz^3^, Dre Radhika Sood^4^, Dr Stijn Bex^1,2^, Dr Christophe Marti^1^, Prof. Jean Luc Reny^1^, Dr Olivier Grosgurin^1,2^*, Dr Antonio Leidi^1,2^*

^1^Division of General Internal Medicine, Department of Medicine, Geneva University Hospitals, Geneva, Switzerland

^2^Division of Emergency Medicine, Department of Acute Care Medicine, Geneva University Hospitals, Geneva, Switzerland

^3^Division of Primary Care Medicine, Geneva University Hospitals, Geneva, Switzerland.

^4^Division of Pneumology, Department of Medicine, Geneva University Hospitals, Geneva, Switzerland.

*These authors contributed equally to the manuscript

Pubmed

("Learning"[MeSH Terms:noexp] OR "education/methods"[MeSH Terms:noexp] OR "teaching/education"[MeSH Terms:noexp] OR "mentoring/methods"[MeSH Terms] OR "internal medicine/education"[MeSH Terms] OR "Learning"[Title/Abstract] OR "Education"[Title/Abstract] OR "Teaching"[Title/Abstract] OR "Training"[Title/Abstract] OR "Curriculum"[Title/Abstract]) AND ("Ultrasonography"[MeSH Terms] OR "Ultrasonics"[MeSH Terms] OR "Echocardiography"[Mesh:NoExp] OR "Ultrasonography"[Title/Abstract] OR "ultrasound"[Title/Abstract] OR "sonography"[Title/Abstract] OR Echography[Title/Abstract] OR Echocardiograph*[Title/Abstract] OR "POCUS"[Title/Abstract]) AND ("randomized controlled trial"[Publication Type] OR "Randomized Controlled Trial" [Publication Type] OR "randomized controlled trials as topic"[MeSH Terms] OR "random allocation"[MeSH Terms] OR "randomized Controlled Trial "[Title/Abstract] OR "RCT"[Title/Abstract] OR "randomly"[Title/Abstract] OR "trial"[Title/Abstract] OR "groups"[Title/Abstract])

EMBASE

('learning'/exp OR 'education'/exp OR 'training'/exp OR 'teaching'/de OR 'mentoring'/de OR ((('internal medicine' OR emergency OR 'general surgery' OR radiology) NEAR/2 (learning OR education OR training OR curriculum OR mentoring)):ab,ti,kw) OR 'computer simulation':ab,ti,kw OR 'virtual reality':ab,ti,kw OR mentoring:ab,ti,kw OR simulation:ab,ti,kw OR curriculum:ab,ti,kw) AND ('ultrasound'/exp OR 'echography'/exp OR 'echocardiograph'/de OR 'point of care testing'/exp OR 'point of care ultrasound'/de OR 'focused assessment with sonography for trauma'/de OR 'point of care system'/de OR 'point-of-care testing':ab,ti,kw OR 'point of care system':ab,ti,kw OR 'ultrasonography':ab,ti,kw OR ultrasound:ab,ti,kw OR sonography:ab,ti,kw OR echography:ab,ti,kw OR echocardiograph*:ab,ti,kw OR 'pocus':ab,ti,kw OR 'focused assessment with sonography for trauma':ab,ti,kw OR ultrasonics:ab,ti,kw) AND ('randomized controlled trial'/de OR 'randomization'/de OR 'single blind procedure'/de OR 'double blind procedure'/de OR 'crossover procedure'/de OR 'placebo'/de OR 'prospective study'/de OR (('randomi?ed controlled' NEXT/1 trial*):ab,ti) OR rct:ab,ti OR 'randomly allocated':ab,ti OR 'allocated randomly':ab,ti OR 'random allocation':ab,ti OR randomized:ti,ab)

WOS

TS=(("learning" OR "education" OR "training" OR "teaching" OR "mentoring" OR (("internal medicine" OR "emergency" OR "general surgery" OR "radiology") NEAR/2 ("learning" OR "education" OR "training" OR "curriculum" OR "mentoring")) OR "computer simulation" OR "virtual reality" OR mentoring OR simulation OR curriculum)) AND TS=(('ultrasound' OR "echography" OR "echocardiograph" OR "point of care testing" OR "point of care ultrasound" OR "focused assessment with sonography for trauma" OR "point of care system" OR "point-of-care testing" OR "point of care system" OR "ultrasonography" OR "ultrasound" OR "sonography" OR "echography" OR "echocardiograph*" OR "pocus" OR "focused assessment with sonography for trauma" OR "ultrasonics")) AND TS=(("randomized controlled trial" OR "randomization" OR "single blind procedure" OR "double blind procedure" OR "crossover procedure" OR "placebo" OR "prospective study" OR (("randomi?ed controlled" NEAR/1 trial*)) OR "rct" OR "randomly allocated" OR "allocated randomly" OR "random allocation" OR randomized))

Cochrane

((("internal medicine" OR emergency OR "general surgery" OR radiology) NEAR/2 (learning OR education OR training OR curriculum OR mentoring)):ab,ti,kw OR "computer simulation":ab,ti,kw OR "virtual reality":ab,ti,kw OR mentoring:ab,ti,kw OR simulation:ab,ti,kw OR curriculum:ab,ti,kw) AND ("point-of-care testing":ab,ti,kw OR "point of care system":ab,ti,kw OR ultrasonography:ab,ti,kw OR ultrasound:ab,ti,kw OR sonography:ab,ti,kw OR echography:ab,ti,kw OR echocardiograph*:ab,ti,kw OR 'pocus':ab,ti,kw OR 'focused assessment with sonography for trauma':ab,ti,kw OR ultrasonics:ab,ti,kw)

ERIC

(DE ("Medical Education" OR "Graduate Medical Education" OR "Nursing Education" OR "Pharmaceutical Education" OR "Medical School Faculty" OR "Medical Schools" OR "Medical Students" OR "Premedical Students" OR "Foreign Medical Graduates" OR "Graduate Medical Education" OR "Learning" OR "Active Learning" OR "Adult Learning" OR "Associative Learning" OR "Aural Learning" OR "Authentic Learning" OR "Cooperative Learning" OR "Discovery Learning" OR "Discrimination Learning" OR "Electronic Learning" OR "Experiential Learning" OR "Game Based Learning" OR "Incidental Learning" OR "Intentional Learning" OR "Interference (Learning)" OR "Lifelong Learning" OR "Mastery Learning" OR "Multisensory Learning" OR "Nonverbal Learning" OR "Observational Learning" OR "Organizational Learning" OR "Prior Learning" OR "Problem Based Learning" OR "Rote Learning" OR "Second Language Learning" OR "Sequential Learning" OR "Serial Learning" OR "Situated Learning" OR "Social Emotional Learning" OR "Student Centered Learning" OR "Symbolic Learning" OR "Transfer of Training" OR "Transformative Learning" OR "Verbal Learning" OR "Visual Learning" OR "Workplace Learning" OR "Educational Methods" OR "Classroom Techniques" OR "Educational Strategies" OR "Psychoeducational Methods" OR "Teaching Methods" OR "Educational Practices" OR "Educational Technology" OR "Methods Research" OR "Hospitals" OR "Mentors" OR "Internal Medicine") OR TI ("learning" OR "education" OR "teaching" OR "training" OR "curriculum" OR "physician assistant programs") OR AB ("learning" OR "education" OR "teaching" OR "training" OR "curriculum" OR "physician assistant programs") OR KW ("learning" OR "education" OR "teaching" OR "training" OR "curriculum" OR "physician assistant programs")) AND  (TI ("Ultrasonography" OR "Ultrasonics" OR "Echocardiography" OR "Ultrasonography" OR "ultrasound" OR "sonography" OR Echography OR Echocardiograph* OR "POCUS" OR point of care bed*) OR AB ("Ultrasonography" OR "Ultrasonics" OR "Echocardiography" OR "Ultrasonography" OR "ultrasound" OR "sonography" OR Echography OR Echocardiograph* OR "POCUS" OR point of care bed*)) AND   (DE ("Randomized Controlled Trials" OR "Comparative Analysis" OR "Control Groups" OR "Evidence" OR "Evidence Based Practice" OR "Experimental Groups" OR "Experimental Programs" OR "Feasibility Studies" OR "Matched Groups" OR "Medical Research" OR "Research Projects" OR "Scientific Research") OR KW ("randomized controlled trial*" OR "Randomized Clinical Trials""random allocation" OR RTC OR Random* OR Trial* OR Group*) OR TI ("randomized controlled trial*" OR "Randomized Clinical Trials""random allocation" OR RTC OR Random* OR Trial* OR Group*) OR AB ("randomized controlled trial*" OR "random allocation" OR RTC OR Random* OR Trial* OR Group*))
