# Supplementary material for: Methods for teaching postgraduate point-of-care ultrasonography: a systematic review
Source: Intern Emerg Med. 2026 Sep 29;21(8):3101–10. doi: 10.1007/s11739-026-04535-3 (PMC13751701; doi:10.1007/s11739-026-04535-3)
Supplement: Supplementary file 4 [file 11739_2026_4535_MOESM3_ESM.docx]

Table 1: PICO elements

| PICO elements | Description |
| --- | --- |
| Population (P) | Include: Physicians (no experience or gender restrictions). Exclude: Medical students, veterinarian |
| Intervention (I) | Include: Any POCUS teaching method (material, instructor, device); teaching defined as learning to acquire and/or interpret images.  Exclude: Ultrasonography teaching of specialists (e.g. echocardiography for cardiologists). Procedural POCUS (vascular access, punction, neurological access) |
| Comparator (C) | No restriction |
| Outcome (O) | Include: Performance (knowledge or technical skill acquisition/improvement), skills retention, trainees’ satisfaction. Exclude: No primary outcome measured. |
| Study design | Include: Randomized (controlled) trials. Exclude: Nonrandomized observational studies. Abstract only |
| Restrictions | No date limit. No language restrictions |

Abbreviations: POCUS, Point-of-care ultrasound

Table 2: Number of studies according to the type of POCUS. eFAST/FAST: extended/Focused Assessment with Sonography for Trauma. RUSH: Rapid Ultrasound for Shock and Hypotension.

| Type of POCUS | Studies (N= 37) | % of total |
| --- | --- | --- |
| FAST/eFAST | 10 [8,10,13,18,20,23,25,33,41,42] | (27%) |
| Multiple organs | 10  [12,14,21,28–30,34,38,39,43] | (27%) |
| Heart | 7 [9,11,15,17,27,36,40] | (18.9%) |
| Lung | 5 [16,19,22,24,32] | (13.5%) |
| Abdomen | 2 [31,44] | (5.4%) |
| Shock/RUSH | 2 [35,37] | (5.4%) |
| Eye | 1[26] | (2.7%) |

Table 3: Simulation-based training

| Study | Specialty | Total sample size (N): group sizes (n) separated by “/” | POCUS type | Study summary | Duration | Assessment and timing of assessment | Outcome |
| --- | --- | --- | --- | --- | --- | --- | --- |
| Paddock et al. [10] | Mixed team | 36: 12 / 12 / 12 | FAST | Traditional with only live patient or only simulator or combined (simulator + live patient) skill training groups, same theoretical content | Total duration not specified; approximately 15–30 minutes (min) of practical training | Pre-intervention MCQ, immediate post-intervention theoretical and practical tests (healthy volunteers) | **Practical**: Mean (± SD) scores were 3.2 / 5 (traditional), 3.3 / 5 (simulator), and 3.3 / 5 (combined).  **Theoretical**: 76 ± 6%, 77 ± 6 %, and 81 ± 6%, respectively, with no statistically significant difference (p>0.05). |
| Edrich et al. [9] | Anesthesia | 46: 23 / 23 | Heart | Theoretical teaching (written and video tutorial) followed by hands-on instruction either on a live model or a simulator | Total duration 80 min including 20-25 min of practical | Written and practical pre-test, immediate post-training written and practical tests, written retention test 4 weeks after training | Simulator training was not inferior to live training.  **Practical**: improved significantly after training in both groups (+4.0 (live) and +4.3 (simulator) points out of 15).  **Theoretical**: Written test performance improved immediately after training in both groups and declined similarly at 4-week follow-up |
| Wilkinson et al. [11] | Internal medicine | 24: 13 (traditional) / 11 (technology) | Heart | Traditional peer-to-peer hands-on teaching versus technology-driven arm combining e-learning and simulator-based practice, also supervised by peer teachers. Both groups had access to a handheld ultrasound device | Total duration 4 hours (h) in a 4-week period, practical time not specified | Pre-test quiz (interpretation) and post-test OSCE with pathological cases | **Theoretical**: similar between groups.  **Diagnostic accuracy** improved in both (conventional +20%, p < 0.001; technology +24%, p = 0.001). False-positive rates increased slightly (conventional +14%, p = 0.079; technology +17%, p = 0.008). |
| Silva et al. [12] | Internal medicine | 40: 19 (live model only) / 21 (simulator) | FAST, head and neck, abdominal, cardiac, pulmonary, musculoskeletal, and some procedural skills | Traditional training with hands-on practice on live models only versus traditional training integrating simulator practice for one of the two practical hours. | 24-h course including 16 h of practical training, over one month | Immediately post-study: written MCQ and practical exam on live model. | No significant difference between groups.  **Practical:** both groups scored 8.7 / 16 (p > 0.05).  **Theoretical**: (simulator) 23.1 ± 3.4 vs. (live model): 21.8 ± 4.8 (p > 0.05). |
| Salen et al[8] | Emergency | 30: 15 / 15 | FAST | Crossover design comparing theoretical presentation followed by simulator or live patient (peritoneal dialysis patient) skill training. | 4-h course including 3 h of practical training | Post-study interpretation test: identify intraperitoneal free fluid on 25 FAST images + satisfaction Likert scale. | No significant difference between groups.  **Theoretical**: 82% ± 7 (live) vs. 78% ± 8 (simulator), p = 0.95. **Satisfaction**: 3.85 vs. 3.25 on 4-point Likert scale, p = 0.317. |
| Juo et al. [13] | General surgery | 16: 8 / 8 | FAST | Theoretical session followed by simulator-only skill training versus live-model (ascitic patients) training | 2-h course including 1 h of practical training. | Pre-test MCQ and immediate post-test: written MCQ + practical exam on live patient. | **Practical**: superior in the live patient group (6/8 vs 1/8 obtaining all four FAST views, p = 0.01)  **Theoretical:** scores improved significantly in both groups (simulator: 21.5 ± 1.6 → 54.2 ± 1.5; live patient: 20.8 ± 2.4 → 52.6 ± 1.2), with no significant difference between groups (p = 0.40). |
| Frère et al.[14] | Anesthesia, intensive care, emergency | 30: 15 / 15 | Focused cardiac ultrasound (FoCUS) and FAST | Both groups received theoretical and clinical practice; the simulator group had additional monthly 1-h self-training sessions on the simulator over 8 months. | 8-month training program. | Theoretical MCQ and practical test on healthy live patients at 8 months. | **Practical:** significantly higher in the simulator group (total score 104 [91–122] vs 83 [69–102]; p = 0.029). Image acquisition also improved (70 [65–81] vs 49 [35–59]; p = 0.0005).  **Theoretical**: no significant difference (32 [24–39] vs 40 [32–44]; p = 0.19). |
| Blanchetière et al. [17] | Pediatric | 52: 17 (control) / 35 (simulation) | Heart | Standard online and bedside NICU rotation training versus same program plus a 2.5–3 h simulator session using the modified Peyton four-step method (demonstration, deconstruction, comprehension, performance) | 6-month rotation, including a single 2.5–3 h simulation session. | Evaluation at 3 and 6 months: practical test (with reference and custom score) on simulator. | **Practical**: Simulation group scored significantly higher at 3 months (reference score: 11.5 ± 2.3 vs 7.4 ± 3.4; p < 0.001; custom score: 25.8 ± 5.3 vs 16.9 ± 7.8; p < 0.001) and at 6 months (11.95 ± 2.46 vs 9.19 ± 3.00; p = 0.003). **Satisfaction:** higher in the simulation group (23.6 ± 2.0 vs 20.4 ± 4.0; p = 0.004). |
| Pietersen et al.[16] | Multiples specialties | 66: 23 (simulation) /22 (live patient) / 21 (control) | Thoracic Ultrasound | Same theoretical online session followed by unsupervised simulator-based or live volunteer training, compared to control (no hands-on). | Duration not specified, 2-h practical session (control: no hands-on). | Post-theory: written test. Post-practical: OSAUS performance assessment on live patients. | **Practical and theoretical:** No significant difference between simulator and live training groups (45.1 vs 41.9 points; respectively, p = 0.38). Both trained groups outperformed the control (36.7 points; p = 0.009). |
| Neelankavil  et al.[15] | Anesthesia | 59: 30 (simulation) / 29 (video) | Heart | Traditional simulator-based cardiac ultrasound training compared with video lectures only, without hands-on practice. | 75 min total including 30 min of practical training for the simulation group versus 45 min video time and no practical training for the video group | Written posttest (20 questions) and practical echocardiography exam on a live patient conducted immediately after training. | **Theoretical**:  The simulation group scored significantly higher on all performance metrics: written test(68.2% ±10.1 vs 57.9% ±8.8; p < .001), image quality (12.4 ±4.2 vs 6.4 ±3.5; p = .003), anatomy identification (17.8 ±6.6 vs 8.3 ±6.6; p = .003), **practical:** simulation group scored higher (78% ±21 vs 50% ±19; p < .001). |

Abbreviations: FAST: Focused Assessment with Sonography in Trauma; MCQ: multiples choice questions; OSCE: Objective Structured Clinical Examination; NICU: neonatal intensive care unit; OSAUS: [Objective Structured Assessment of Ultrasound Skills](https://www.google.com/search?q=Objective+Structured+Assessment+of+Ultrasound+Skills&client=safari&hs=EuJU&sca_esv=7a0616c681a8c1ce&rls=en&biw=1440&bih=820&sxsrf=AE3TifMEA-bglkVCBlFe_O5kqGxZtvQqog%3A1765873609787&ei=yRdBac3uL_a5i-gP84Gc6As&ved=2ahUKEwig9OLs18GRAxW22QIHHTT0F5QQgK4QegQIARAE&uact=5&oq=OSAUS+abbrevaition+medical+ultrasound&gs_lp=Egxnd3Mtd2l6LXNlcnAiJU9TQVVTIGFiYnJldmFpdGlvbiBtZWRpY2FsIHVsdHJhc291bmQyBxAhGKABGApIwQxQwwFY6AtwAXgBkAEBmAGeAqABlg6qAQUwLjcuM7gBA8gBAPgBAZgCCqACiw3CAgoQABiwAxjWBBhHwgIGEAAYDRgewgIIEAAYCBgNGB6YAwDiAwUSATEgQIgGAZAGCJIHBTEuNi4zoAezLrIHBTAuNi4zuAeHDcIHBTIuNS4zyAcXgAgA&sclient=gws-wiz-serp)

Table 4: Web based training

| Study | Specialty | Total sample size (N): group sizes (n) separated by “/” | POCUS type | Study summary | Duration | Assessment and timing of assessment | Outcome |
| --- | --- | --- | --- | --- | --- | --- | --- |
| Soon et al.[24] | Pediatric | 45: 23 (traditional) / 22 (e-learning) | Lung | Traditional classroom lecture with live model practice versus identical content web-based tutorial with live model practice | 40 min total (30 min practical) | Knowledge test after theoretical introduction; OSCE after hands-on training; survey at the end; repetition after 2 months (immediate and 2-month follow-up) | No significant differences between classroom and web groups.  **Practical** OSCE tests (95% CI pre−2.5 [−12 to 6.9]; post 2.0 [−1.4 to 5.3]; retention −0.8 [−9.6 to 8.1]). OSCE post 1.9 [−4.7 to 8.5], retention−0.6 [−10.7 to 9.5]. Web-based learning is more time-efficient |
| Haskins et al. [21] | Anesthesia | 18: 9 / 9 | LUS and FAST | Traditional didactic lecture with hands-on practice versus identical e-learning module with hands-on practice | 2 h (traditional), 2.5 h median time on e-learning, practical time not specified. | Theoretical and image acquisition tests conducted pre, post, and 5 months safter training | **Practical**: no difference post-hands-on practical test scores (−4.2 [−24.6, 16.3]; P = .672).  **Theoretical**: no difference between e-learning and didactic groups in the change in LUS knowledge (difference in median percentage point change [95% CI]: 6.6 [−10.0, 23.2]; P = .411). Posttest LUS knowledge scores were also similar (difference [95% CI]: −0.9 [−4.8, 3.0]; P = .629), as were FAST knowledge scores (0; P = .999)  **Satisfaction** did not differ between groups |
| Isa et al. [25] | None (junior doctors) | 33: 16 (video) / 17 classroom) | FAST | Self-directed video-based theoretical and skill learning using simulated patients versus classroom lecture and supervised hands-on training with facilitator supervision | 4 h total including self-practice (duration not specified) | OSCE and post-test satisfaction questionnaire | The self-instructional video method was non-inferior to classroom teaching for learning FAST. **Practical**: (mean 35.6 ± 5.9 vs 35.3 ± 3.4; p = 0.871), with an absolute difference of 0.3 (95% CI −3.75 to 3.21). **Satisfaction**: Video group reported lower confidence in performing FAST. |
| Monti et al. [23] | Military (US Marines) | 34: 17 / 17 + 20 emergency medicine residents (EM) (comparison) | eFAST | Web video lecture and supervised hands-on training versus traditional lecture and supervised hands-on training, results compared to those of 20 EM residents who didn't do the training. | 4 h total including 150 min of practical training. | 50 MCQ pretest and post-test; OSCE conducted on live and phantom models immediately after the course. | **Practical**: No significant differences between web and classroom groups Mean OSCE:20.9 (IQR 21–22) vs 20.6 (IQR 20–22), p = 0.33;  **Theoretical**: mean written post-course score: 84% (78–92) vs 82% (78–86), p = 1.00. |
| Morales-Castro et al. [27] | Internal medicine | 28: 14 /14 | Heart | Both groups received didactic teaching and simulator-based training; the intervention group had access to self-learning echocardiography videos, while the control group used written materials only. | 2 h total (practical time not specified) | Assessment included 10 MCQs and a practical simulator test conducted at baseline, 1 month, and 6 months post-training. | Both groups significantly improved post-training. **Practical**: no difference: image quality (33% vs 20%, p = 0.6), or comfort level (31% vs 25%, p = 0.7). Scan time was significantly shorter in the video group (72 s vs 38 s, p = 0.02).  **Theoretical**: no differences (23% vs 19%, p = 0.7)  Limited video use precluded firm conclusions. |
| Nilsson et al. [20] | None (newly graduated interns) | 34: 16 mobile app / 18 (textbook) | eFAST | Mobile app-guided self-directed training versus textbook-guided self-directed training. | 2 h total including 2 h of self-practice. | Theoretical and OASUS practical test on simulated patients (one with peritoneal fluid) performed 2 weeks after training. | **Practical**: Mobile app-guided group: mean OSAUS score 42.3% (95% CI 38.5–46.0) vs textbook-guided 45.3% (95% CI 39.3–51.3); p = 0.41.  **Theoretical:** median correct answers 36 (IQR 2.44) vs 37 (IQR 4.0); p = 0.163. Mobile app training was less cost-effective. |
| Platz et al.[18] | Multiple specialties | 55: 18 (web)/ 13 (classroom) / 16 (control) | eFAST | Web-based lectures followed by a practical workshop session versus classroom course with identical lectures and hands-on, control without any education. | 8 h for the classroom course versus 4 h for the web-based course; duration of practical training not specified. | Knowledge evaluated through MCQ pre-test, post-test, and 8-week follow-up test. | **Theoretical**: Both web-based and classroom groups showed significant improvement in knowledge post-training (web 77.8% → 92.5%; classroom 75.9% → 93.9%; p < .001) with similar retention after 8 weeks (88.6% vs 88.9%; p = .87). No significant difference between the two modalities (Δ < 2%).  The control group showed no learning effect without intervention (83.3% versus 82.8%, P .88). |
| Kang et al.[26] | Emergency | 32: 16 /16 | Eye | Online ocular ultrasound training without hands-on practice versus classroom-based didactic lecture with a supervised hands-on session. | 30 min individual hands on for the traditional group versus 20 min of videoclip (online group) | Pre-survey and ocular scan before training; immediate post-training knowledge test + post-survey; repeat ocular scan 2 weeks later. | **Practical and theoretical:** Total score (mean ± SE):21.7 ± 0.35 vs 21.3 ± 0.25; difference 0.35 ± 0.42 (95 % CI −0.48 to 1.17). No difference in scan time or knowledge retention; higher satisfaction reported in the hands-on group (p < 0.001). |
| Edrich et al.  [19] | Anesthesia | 138: 59 (traditional) / 59 (web) / 20 (control) | Lung | Narrated online video with self-performed lung ultrasound compared with classroom lecture and supervised hands-on training of identical content; a control group received no education. | 65 min (classroom) vs 30 min (web), including 20 vs 5 min of practical training. | Written MCQ and practical exam administered pre-training, post-training, and at 4-week retention. | **Practical and theoretical:** Both the classroom and web groups showed significant improvement in test performance (mean increase +42.9 % vs +39.2 %; P = 1.00), while the control group did not (+1.2 %). The difference between web and classroom groups (−3.7 [95 % CI −10.6 to 3.2]) was within the non-inferiority margin. Web-based training was also more time-efficient. |
| Tomasi et al. [22] | Anesthesia | 64: 23 (traditional) / 23 (web) / 8 (control) | Lung | Three training groups were compared: web-based video learning with self-scanning, traditional lecture with supervised practical sessions, and a control group with no education. | 50 min (traditional), 40 min (web), and 0 min (control); practical duration 20, 15, and 0 min respectively. | Satisfaction and self-evaluation questionnaires completed immediately post-training. | **Satisfaction**: Both web-based and classroom training were rated equally. Course quality was higher for classroom (78.3% vs 39.1%, p < 0.05). Need for further training differed (Online: 21.7%; Classroom: 60.9%; Control: 62.5%; p < 0.05). LUS mainly used for pleural effusion (41.3%) and pneumothorax (32.6%). Hybrid teaching preferred (Online: 52.2%; Classroom: 56.5%; Control: 62.5%). |

Abbreviations: LUS: Lung ultrasound; FAST: Focused Assessment with Sonography in Trauma; eFAST: Extended Focused Assessment with Sonography in Trauma; OSCE: Objective Structured Clinical Examination; OSAUS: [Objective Structured Assessment of Ultrasound Skills](https://www.google.com/search?q=Objective+Structured+Assessment+of+Ultrasound+Skills&client=safari&hs=EuJU&sca_esv=7a0616c681a8c1ce&rls=en&biw=1440&bih=820&sxsrf=AE3TifMEA-bglkVCBlFe_O5kqGxZtvQqog%3A1765873609787&ei=yRdBac3uL_a5i-gP84Gc6As&ved=2ahUKEwig9OLs18GRAxW22QIHHTT0F5QQgK4QegQIARAE&uact=5&oq=OSAUS+abbrevaition+medical+ultrasound&gs_lp=Egxnd3Mtd2l6LXNlcnAiJU9TQVVTIGFiYnJldmFpdGlvbiBtZWRpY2FsIHVsdHJhc291bmQyBxAhGKABGApIwQxQwwFY6AtwAXgBkAEBmAGeAqABlg6qAQUwLjcuM7gBA8gBAPgBAZgCCqACiw3CAgoQABiwAxjWBBhHwgIGEAAYDRgewgIIEAAYCBgNGB6YAwDiAwUSATEgQIgGAZAGCJIHBTEuNi4zoAezLrIHBTAuNi4zuAeHDcIHBTIuNS4zyAcXgAgA&sclient=gws-wiz-serp)

Table 5: Tele-mentoring

| Study | Specialty | Total sample size (N): group sizes (n) separated by “/” | POCUS type | Study summary | Duration | Assessment and timing of assessment | Outcome |
| --- | --- | --- | --- | --- | --- | --- | --- |
| Mao et al. [41] | General surgery | 14: 7 / 7 | FAST | Remote instruction (presentation + hands-on on simulator) supervised via tele-mentoring versus in-person instruction with identical content. | NA | Pre-test: survey + practical exam on a mannequin (presence of free fluid). Immediately post-course: confidence survey. Six months post-study: repeat practical exam + follow-up survey. | **Practical**: No significant difference between groups. Both groups improved at six months (remote 85 ± 13.7 %, in-person 87.5 ± 13.7%, NS).  **Confidence**: Post-course increased in both groups (remote 3.12 → 4.13; in-person 3.17 → 4.02; NS). |
| Kim et al. [44] | Multiple specialties | 40: 20 / 20 | Abdomen | Video lecture followed by hands-on training under mixed reality-based tele-mentoring supervision versus traditional in-person supervision by faculty. | 15 min of theoritical content, hands on not specified | Immediately post-study: practical assessment of image quality, workload (NASA-TLX), and satisfaction survey. | **Practical**: No significant difference in image quality (tele supervision group (TG): 66.8 ± 10.3 vs direct supervision group (DG): 66.8 ± 10.4, p = .84) or procedure time (TG 23.8 ± 8.0 vs DG 24.0 ± 8.1 min, p = .95).  **Workload**: TG performed more educational interactions (4.0 ± 2.5 vs 0.8 ± 1.1, p < .001) and reported lower mental demand, effort, and frustration (all p < .05). |
| Gillon et al. [42] | Pediatric | 50: 26 (virtual) / 24 (traditional) | eFAST | Virtual instruction using handheld device with wireless streaming under remote supervision versus traditional in-person instruction. | No time limit, each participant determined when they had received sufficient instruction | Pre-study survey followed by post-training (≤ 7 days) survey and practical EFAST test on standardized model. | **Practical and theoretical:** No significant difference between virtual and in-person instruction. Assessment scores: 65.8% vs 61.8% (p = .349) or assessment duration:482.6 s vs 432.6 s (p = .346). |
| Shen et al. [43] | Critical care | 90: 45 /45 | Abdominal, lung, cardiac | Tele-mentoring real-time interactive traditional training with instructor feedback versus online theoretical-only video training. | NA | Immediate post-test: combined theoretical and practical assessment covering critical care ultrasound topics; satisfaction survey one month later. | **Practical and theoretical:** Significant improvement in the intervention group. Pre-training: experimental:  42.2 ± 12.5 vs control: 41.9 ± 14.3 (p = .907). Post-training: 88.4 ± 6.7 vs 72.9 ± 10.8 (p < .001). Satisfaction higher (exp 42.6 ± 2.3 vs ctrl 34.7 ± 3.1, p < .001). |

Abbreviations: LUS: Lung ultrasound; FAST: Focused Assessment with Sonography in Trauma; eFAST: Extended Focused Assessment with Sonography in Trauma; OSCE: Objective Structured Clinical Examination; OSAUS: [Objective Structured Assessment of Ultrasound Skills](https://www.google.com/search?q=Objective+Structured+Assessment+of+Ultrasound+Skills&client=safari&hs=EuJU&sca_esv=7a0616c681a8c1ce&rls=en&biw=1440&bih=820&sxsrf=AE3TifMEA-bglkVCBlFe_O5kqGxZtvQqog%3A1765873609787&ei=yRdBac3uL_a5i-gP84Gc6As&ved=2ahUKEwig9OLs18GRAxW22QIHHTT0F5QQgK4QegQIARAE&uact=5&oq=OSAUS+abbrevaition+medical+ultrasound&gs_lp=Egxnd3Mtd2l6LXNlcnAiJU9TQVVTIGFiYnJldmFpdGlvbiBtZWRpY2FsIHVsdHJhc291bmQyBxAhGKABGApIwQxQwwFY6AtwAXgBkAEBmAGeAqABlg6qAQUwLjcuM7gBA8gBAPgBAZgCCqACiw3CAgoQABiwAxjWBBhHwgIGEAAYDRgewgIIEAAYCBgNGB6YAwDiAwUSATEgQIgGAZAGCJIHBTEuNi4zoAezLrIHBTAuNi4zuAeHDcIHBTIuNS4zyAcXgAgA&sclient=gws-wiz-serp); NA: Not Applicable

Table 6: Training intensity

| Study | Specialty | Total sample size (N): group sizes (n) separated by “/” | POCUS type | Study summary | Duration | Assessment and timing of assessment | Outcome |
| --- | --- | --- | --- | --- | --- | --- | --- |
| Noble et al. [29] | Emergency medicine | 12: 6 / 6 | FAST, cardiac, aorta, renal, gallbladder, pelvic | Traditional classroom including theoretical and one-on-one proctored hands-on sessions versus theoretical-only session without practical training. | 9.5 h total including1.5 h practical for the intervention group vs 8 h and no practical time for control. | 24 image-based MCQs administered pre-course and repeated at 6 months. | **Theoretical**: Proctored ultrasound group exhibited greater score improvement than untrained peers. Post-test improvement 2.0 (95% CI 1.0–3.0) in controls vs 5.0 (95% CI 3.0–6.0) in intervention; between-group difference 3.0 (95% CI 1.0–5.0), not statistically significant. |
| Matthews et al. [32] | Internal medicine | 28: 14 / 14 | Thoracic ultrasound | Theoretical and practical course plus 20 supervised thoracic ultrasounds(intervention) versus identical theoretical and practical course without supervised practice (control). | 14 h total including 6 h of practical; intervention group received 20 additional supervised scans. | Written and practical exams conducted pre-course, immediately post-course, and at 6 and 12 months. | **Practical**: Intervention group achieved significantly higher exam scores at 6 months (median 82.61 [72.83–89.13] vs 63.05 [48.92–69.57]; p < 0.001). **Theoretical**: Written scores improved for all participants without significant difference. |
| Ramsingh et al.[30] | Anesthesia | 20: 10 / 10 | Cardiopulmonary, volume status, trauma ultrasound | Traditional classroom with theoretical and practical content on a simulator or human model versus theoretical-only session (no hands-on). | 1.5 h total, no practical training in control group. | MCQ and simulator practical test conducted pre-course and immediately post-course. | **Practical**: Traditional group scored significantly higher on simulator exam (median 18 [14.3–21.3] vs 11.5 [8–15.8], p = 0.041).  **Theoretical**: Traditional group scored significantly higher on MCQ (median 19.5 [16.8–20.8] vs 15.5 [13.3–18.8], p = 0.038) |
| Todsen et al. [31] | Multiple specialties (mostly primary care) | 31: 17 (traditional) / 14 (control) | Abdominal | Traditional classroom training with theoretical and hands-on components versus no education. | 4 h total (intervention group). | OASUS test performed on live patients with pathological findings at the end of the study, outcome was diagnostic accuracy | **Practical**: Intervention group had significantly higher performance (27.4% vs 18.0%, p = 0.004) and diagnostic accuracy (65% vs 39%, p = 0.014). |
| Liu et al. [35] | Emergency medicine | 100: 50 / 50 | Shock assessment | Multimodal strategy (visualization+ mind mapping + problem-oriented learning) including theory and practice versus traditional classroom teaching. | NA | Theoretical and practical tests conducted immediately after training. | **Practical**: Multimodal group scored higher in practical test (82.3 ± 7.4 vs 68.2 ± 9.5, p < 0.001)  **Theoretical**: Multimodal group scored higher in theory (80.6 ± 7.9 vs 60.6 ± 11.7, p < 0.001)  **Satisfaction**: higher satisfaction, engagement, and self-learning with multimodal group (p < 0.001). |
| Leidi et al.[28] | Internal medicine | 23: 11 (structured tutoring) / 12(control) | Heart and lung ultrasound | Traditional teaching followed by structured tutoring (scheduled paired supervision sessions over 6 months) versus traditional classroom and voluntary supervision. | 6-month duration with bedside supervision. | Theoretical (MCQ), OSCE, and number of supervised scans evaluated after 6 months. | **Practical**: Structured tutoring increased supervised scans (median 27 vs 0), ≥25 scans (70% vs 0%, p < 0.001), and autonomy (83% vs 33%, p = 0.002); improved heart imaging skills (p < 0.05), no difference for lung imaging. |
| Suzuki et al.[34] | Critical care | 21: 11 (intensive) / 10 (control) | Cardiac, thoracic, abdominal, vascular | Traditional course followed by bedside training, e-learning, lecture and intensive training (1 h standardized patient, 6 h bedside, 1 h interpretation) versus traditional training followed by bedside training, e-learning and lecture only. | 2 half days followed by 6-month program; intensive arm added 8 h. | Written and hands-on proficiency test conducted pre-course and immediately post-course. | **Practical**: No significant difference in proficiency improvement: median (IQR) 31 (21–46) vs 18 (3.8–38), p = 0.09. |
| Smith et al.[33] | Emergency medicine | 16: 8 / 8 | eFAST | Traditional training plus mastery learning (feedback and retesting until achievement of predefined proficiency score) versus traditional lecture, cross-over after 6 months. | Duration not specified (≈ 25 min theoretical). | Practical exam (53-item checklist) conducted every 3 months over 12 months (pre-, mid-, and post-training intervals). | **Practical**: Mastery group: higher proficiency earlier. Interval 1: 79 [75–83] vs control 73 [69–78] (p = 0.09); Interval 2: 80 [76–84] vs 73 [69–77] (p = 0.013). No differences there after (p > 0.3). Both groups reached MPS by year-end. |

Abbreviations: LUS: Lung ultrasound; FAST: Focused Assessment with Sonography in Trauma; eFAST: Extended Focused Assessment with Sonography in Trauma; OSCE: Objective Structured Clinical Examination; OSAUS: [Objective Structured Assessment of Ultrasound Skills](https://www.google.com/search?q=Objective+Structured+Assessment+of+Ultrasound+Skills&client=safari&hs=EuJU&sca_esv=7a0616c681a8c1ce&rls=en&biw=1440&bih=820&sxsrf=AE3TifMEA-bglkVCBlFe_O5kqGxZtvQqog%3A1765873609787&ei=yRdBac3uL_a5i-gP84Gc6As&ved=2ahUKEwig9OLs18GRAxW22QIHHTT0F5QQgK4QegQIARAE&uact=5&oq=OSAUS+abbrevaition+medical+ultrasound&gs_lp=Egxnd3Mtd2l6LXNlcnAiJU9TQVVTIGFiYnJldmFpdGlvbiBtZWRpY2FsIHVsdHJhc291bmQyBxAhGKABGApIwQxQwwFY6AtwAXgBkAEBmAGeAqABlg6qAQUwLjcuM7gBA8gBAPgBAZgCCqACiw3CAgoQABiwAxjWBBhHwgIGEAAYDRgewgIIEAAYCBgNGB6YAwDiAwUSATEgQIgGAZAGCJIHBTEuNi4zoAezLrIHBTAuNi4zuAeHDcIHBTIuNS4zyAcXgAgA&sclient=gws-wiz-serp); NA: Not Applicable

Table 7: Other methods

| Study | Specialty | Total sample size (N): group sizes (n) separated by “/” | POCUS type | Study summary | Duration | Assessment and timing of assessment | Outcome |
| --- | --- | --- | --- | --- | --- | --- | --- |
| Baum et al. [40] | Internal medicine | 43: 22 (AI) / 21 no AI) | Heart | Handheld ultrasound with artificial intelligence guidance versus handheld ultrasound without AI guidance for 2 weeks, self-practice without further teaching. | 2 weeks study time | Practical test performed at the end of the study (2 weeks): time to acquire image on a live patient + RACE score. | **Practical**: AI group: faster scan times (72 s [IQR 38–85]) vs non-AI (85 s [IQR 54–166]), p = 0.01; higher image quality (4.5 [IQR 2–5.5]) vs 2.0 [IQR 1–3], p < 0.01; correct systolic function identification 85% vs 50%, p = 0.02.  **Confidence**: no difference. |
| Hofer et al. [39] | NA | 90: 47 (peer-teacher) / 43 (traditional) | Abdominal and emergency ultrasound | Theoretical and ultrasound course, same content, practical session (quiz and hands-on) taught by either student instructors versus physician instructors. | 17.25 h total including13.5 h practical. | OSCE and satisfaction survey administered immediately post-training. | **Practical**: Peer-teacher group: mean OSCE 76.4/100 vs physician-instructor 73.7/100 (p = 0.28). Didactic skills levels: students 1.44, residents 1.46, attendings 1.29. **Satisfaction**: didactic competence: 1.53 vs 1.57 vs 1.53, no significant difference. |
| Buesinget al.[38] | Internal medicine | 47: 24 (HUD / 23 (no-HUD) | Thoracic, echocardiography, abdominal | Traditional teaching plus handheld ultrasound device (HUD) for self-practice versus traditional instruction without device. | 8 – 12 h total including 4 – 6 h practical over a year | Pre- and post-training RACE and B-QUIET tests on live subjects + weekly survey throughout the training period. | **Practical**: HUD group performed 6.8 ± 2.2 scans/week vs 6.4 ± 2.9 for controls (p = 0.66). Median RACE (Rapid Assessment of Competency in Echocardiography) score 17.0 vs 17.8 (p = 0.72). No correlation with exam frequency (ρ = 0.022, p = 0.95). No improvement in acquisition ability or use. |
| Lai et al. [37] | None (junior doctors) | 31: 16 (gamified)/ 15 (traditional) | RUSH protocol | Gamified team-based quizzes and point system training versus conventional theoretical and practical training. | 9.1 h total including 3.1 h practical. | Pre- and post-training MCQ + OSCE + 2-month retention test. | **Practical:** No significant difference between gamified and traditional groups in skills F (2,58) = 3.06, p = 0.06).  **Theoretical:**  No significant difference between gamified and traditional groups in knowledge (F (2,58) = 39.6, p<0.001; **Satisfaction**: Gamified group reported higher motivation and engagement. |
| Campanella et al. [36] | Emergency medicine | 35: 16(control)/19 (bovine heart) | Heart | Instructional video and static 2D images versus same materials plus dissected bovine heart model to teach cardiac anatomy (no scanning) | NA | Theoretical test on 24 labelled ultrasound images immediately post-training. | **Theoretical**:  Bovine heart group: mean score improvement+11.3 ± 4.5 vs control +7.6 ± 4.0 (p = 0.016). After baseline adjustment, difference not significant (+1.8, 95% CI −1.1 to 4.8, p = 0.22). |

Abbreviations: LUS: Lung ultrasound; FAST: Focused Assessment with Sonography in Trauma; eFAST: Extended Focused Assessment with Sonography in Trauma; OSCE: Objective Structured Clinical Examination; OSAUS: [Objective Structured Assessment of Ultrasound Skills](https://www.google.com/search?q=Objective+Structured+Assessment+of+Ultrasound+Skills&client=safari&hs=EuJU&sca_esv=7a0616c681a8c1ce&rls=en&biw=1440&bih=820&sxsrf=AE3TifMEA-bglkVCBlFe_O5kqGxZtvQqog%3A1765873609787&ei=yRdBac3uL_a5i-gP84Gc6As&ved=2ahUKEwig9OLs18GRAxW22QIHHTT0F5QQgK4QegQIARAE&uact=5&oq=OSAUS+abbrevaition+medical+ultrasound&gs_lp=Egxnd3Mtd2l6LXNlcnAiJU9TQVVTIGFiYnJldmFpdGlvbiBtZWRpY2FsIHVsdHJhc291bmQyBxAhGKABGApIwQxQwwFY6AtwAXgBkAEBmAGeAqABlg6qAQUwLjcuM7gBA8gBAPgBAZgCCqACiw3CAgoQABiwAxjWBBhHwgIGEAAYDRgewgIIEAAYCBgNGB6YAwDiAwUSATEgQIgGAZAGCJIHBTEuNi4zoAezLrIHBTAuNi4zuAeHDcIHBTIuNS4zyAcXgAgA&sclient=gws-wiz-serp); NA: Not Applicable; RACE : Rapid Assessment of Competency in Echocardiography Scale; B-QUIET: Brightness Mode Quality Ultrasound Imaging Examination Technique; HUD: handheld ultrasound device

Table 8: Medical Specialty of the study participants

| Specialty | Number of studies (%) | Number of participants (%) |
| --- | --- | --- |
| Anaesthesiology | 6 (16.2) [9,15,19,21,22,30] | 345 (22) |
| Multidisciplinary ^a^ | 5 (21.6) [14,16,18,31,44] | 320 (20.4) |
| Internal medicine | 7 (18.9) [11,12,27,28,32,38,40] | 233 (14.8) |
| Emergency medicine | 6 (16.2) [8,26,29,33,35,36] | 225 (14.3) |
| Paediatrics | 3 (8.1) [17,24,42] | 147 (9.4) |
| Critical care | 2 (5,4)  [34,43] | 111 (7) |
| Junior doctor | 3 (8.1) [20,25,37] | 98 (6.2) |
| Not specified | 1 (2.7) [39] | 90 (5.7) |
| Multiprofessional ^b^ | 1 (2.7) [10] | 36 (2.3) |
| Military physicians | 1 (2.7) [23] | 34 (2.2) |
| General surgery residents | 2 (5.4)  [13,41] | 30 (1.9) |

a: including junior doctors, multiples specialties in the same study

b: mixed disaster team comprising doctors, nurses, paramedic

*Assessment*:

Twenty-one studies employed a combined written and practical assessment, pairing multiple choice question (MCQ)-based or image-interpretation tests with structured hands-on performance evaluations on live models or simulators [9,10,12–16,19–21,23,24,26–28,30,32,34,35,37,43].

Practical-only assessments were used in 11 studies, focusing exclusively on image acquisition or diagnostic task performance [11,17,25,31,33,38–42,44].

Written-only evaluations appeared in four trials [8,18,29,36].

The study by Tomasi [22] relied exclusively on self-assessed confidence and user satisfaction questionnaires, without any objective theoretical or practical performance testing, and was therefore not classifiable within the above assessment categories.

Moreover, retention or follow-up assessments were performed in only 16 trials [9,14,18–21,24,26–29,32,33,37,38,41]
